# Performance and quality of eggs of laying hens fed with *Moringa Oleifera* leaf flour

**DOI:** 10.1371/journal.pone.0314905

**Published:** 2024-12-31

**Authors:** Rogério Ventura da Silva Junior, Carlos Bôa-Viagem Rabello, Maria do Carmo Mohaupt Marques Ludke, Cláudia da Costa Lopes, Waleska Rocha Leite de Medeiros Ventura, Elayne de Souza Rocha Soares, Patricia Maria Guedes Paiva, Thiago Henrique Napoleão, Apolonio Gomes Ribeiro, Júlio Cézar dos Santos Nascimento, Lilian Francisco Arantes de Souza, Helia Sharlane de Holanda Oliveira

**Affiliations:** 1 Animal Science Department, Universidade Federal Rural de Pernambuco, Recife, PE, Brazil; 2 Unidade Acadêmica Especializada em Ciências Agrárias, Universidade Federal do Rio Grande do Norte, Distrito de Jundiaí–Macaiba, Rio Grande do Norte, Brazil; 3 Biochemistry and physiology Department, Universidade Federal de Pernambuco, Recife, PE, Brazil; 4 Animal Science Department, Universidade Federal da Paraíba, Areia, PB, Brazil; Tokat Gaziosmanpaşa University: Tokat Gaziosmanpasa Universitesi, TÜRKIYE

## Abstract

The objective of this study was to evaluate the performance of commercial laying hens fed with different levels of *Moringa oleifera* leaf meal in their diet. For this purpose, 150 laying hens of the Dekalb White lineage, at 62 weeks of age, and with an initial average weight of 1.458 kg ± 8.70g, were used. They were housed in cages measuring 1.00 X 0.40 X 0.45m, equipped with chute-type feeders, automatic cup-type drinkers and chute for collecting eggs. They were distributed in a completely randomized design, consisting of five treatments with five replications of six birds. The treatments consisted of a reference diet, without moringa inclusion, and four test diets with levels of 1.5%, 3.0%, 4.5% and 6% inclusion of moringa leaf meal. Water was provided ad libitum, and 120 g of feed per bird per day. The total experimental period lasted 122 days. The data obtained were subjected to analysis of variance and Dunnett’s test at 5% significance and, when significant, subjected to regression analysis. Analysis of the leaf flour revealed the presence of antinutritional factors in small concentrations. It was observed that the addition of moringa to the birds’ diet did not influence performance parameters and provided significant increases in the average egg weight according to moringa inclusion levels of 1.5%, 4.5% and 6% when compared to the control diet, as well as a significant increase in the color of the egg yolks for all levels tested (1.5 to 6%). A reduction in Haugh unit values was observed when inclusion levels of moringa were 4.5% and 6%. Despite a reduction in Haugh unit values, they are still within the classification standards. Analysis of the biochemical parameters of the birds’ blood serum did not show significant influences according to the levels of moringa inclusion in the diet, although a numerical reduction was observed in the cholesterol levels of birds fed with an inclusion of 6% of leaf flour in the diet, which could possibly be related to the presence of phytochemical compounds, more precisely β-sitosterol. *Moringa oleifera* leaf meal can be used to feed laying birds without causing harm to the birds’ performance, in addition to intensifying the color of the yolks.

## Introduction

In the current poultry industry, rising feed ingredient prices have encouraged the search for more economical agricultural by-products [1, 2]. The supply of protein sources has become increasingly limited worldwide, creating the need for viable alternatives [3]. Feed costs represent a significant portion of total expenses, and sudden increases in these costs challenge nutritionists to maintain production and animal welfare while trying to balance diet costs [4]. Soybean meal (SBM), widely used as a protein source in poultry diets, has also faced price increases that prompt nutritionists to seek available ingredients to create cost-effective, nutritionally balanced, and financially viable formulations [2, 5, 6]. In this context, the development of diets with alternative ingredients presents an effective solution to mitigate these costs, especially when such ingredients are locally accessible.

A wide range of plant-based alternative products can be used in poultry feeding, including forage hay, which has been evaluated as having potential for better utilization in poultry diets, and mulberry hay (Morus alba) [7], leucaena hay (Leucaena leucocephala) [8], hay made from the aerial part of cassava (Manihot esculenta Crantz) [9], alfalfa hay (Medicago sativa) [10], and moringa (Moringa oleifera) [8, 11, 12].

Moringa oleifera Lam, a plant belonging to the Moringaceae family, comprises approximately 14 known species. Among its characteristics are high protein content [13], ranging from 18.29 to 31.5% crude protein [8, 14, 15], essential amino acids [15–17], and minerals such as calcium and phosphorus, along with precursors of vitamin A, B-complex vitamins, and vitamin C [15, 18–20]. When provided to laying hens, Moringa oleifera has demonstrated satisfactory effects, significantly improving yolk color, egg production, and egg weight [12, 21], without affecting shell thickness or egg shape index [12].

Research to determine optimal levels of Moringa oleifera in layer hen diets is still not well understood, as moringa is a fibrous feed and contains certain bioactive compounds that, if ingested in large amounts, may be harmful to poultry, including phenolic compounds, tannins, lignin, saponins, phytate, protease inhibitors, oxalates, and cyanogenic glycosides [22–28]. Therefore, the objective of this research was to evaluate the impacts of including Moringa oleifera leaf meal in layer hen diets and its effects on productive performance and egg quality.

## Material and methods

The research was approved by the Ethics Committee on the Use of Animals (CEUA), of the Federal Rural University of Pernambuco, in accordance with license number 085/2015.

### Experiment site, facilities, animals, and management

A performance experiment was conducted in the laying poultry shed belonging to the Bird Research Laboratory located in the Animal Husbandry Department of the Federal Rural University of Pernambuco (UFRPE), in the municipality of Recife, state of Pernambuco, located at 4.5 m altitude in relation to sea level and geographic coordinates 8°3’14’’ S latitude and 34°52’52’’ W longitude.

For the research, 150 laying birds of the Dekalb White lineage were used, aged 62 weeks and with an average weight of 1458 kg ± 8.70 g. They were housed in cages measuring 1.00 X 0.40 X 0. 45m with a chute for collecting eggs, a chute-type feeder, and an automatic drinker with an attached cup. The birds were weighed at the beginning of the experimental period to obtain uniformity between the experimental plots. Then, the birds had their egg production per experimental unit monitored for a period of 14 days. After standardizing egg weight and production, the treatments were randomly distributed through drawings among the experimental units.

The lighting program adopted was 17 hours of light (12 hours of natural light and 5 hours of artificial light), according to the Dekalb White lineage’s manual [29]. The temperature and relative humidity of the air inside the warehouse were recorded daily with a Datalogger. An average temperature of 27.89°C and relative humidity of 79% were recorded throughout the experimental period. The trial lasted 122 days, of which 10 days were used to adapt the birds to the experimental diets, thus composing four cycles of 28 days. Water was provided *ad libitum* and the amount of feed was 120 g/bird/day. The eggs produced were collected twice a day (9 am and 3 pm), counted and weighed per experimental unit, to obtain the average weight. Weekly, leftover feed was collected from the feeders to calculate feed intake.

### Production of *Moringa oleifera* leaf flour

Leaves and petioles of *Moringa oleifera* were collected at intervals of 45 days between plant cuts for use in producing the flour. The plants were cut at a height of approximately 50 cm from the ground. After cutting, the entire production was transported and housed in a warehouse protected against adverse weather conditions (sun and rain). Leaves and petioles were separated from the central stems, and then left in the shed to dry (dried to a constant weight), which produced hay, which was later ground by hammer mill (DPM-4 Forage Grinder—hammer type, 5.0mm Sieve—Nogueira) into leaf flour.

### Bromatological, aminoacidic, and antinutritional factor analyses

Samples of the leaf flour produced were sent to the Animal Nutrition Laboratory (LNA) of the Department of Animal Science at UFRPE to determine the contents of dry matter, crude protein, neutral detergent fiber, acid detergent fiber, ether extract, and mineral matter according to the methodology proposed by Detmann et al. [30]. Gross energy (EB) was determined in a calorimetric pump IKA-C 200 model. A sample of the flour was sent to the EVONIK® company laboratory for aminoacidic analysis using the protein hydrolysis followed by HPLC (High Performance Liquid Chromatography) reading. The values determined after the analyses are shown in Table 1.

**Table 1:** Bromatological, energetic, and aminoacidic composition of *Moringa oleifera* (MS) leaf flour.

Nutrients				
Metabolizable Energy^1^, kcal/kg	1,980		Total amino acids^3^, %	
Brute Energy, kcal/kg	3,967		Methionine	0.306
Dry Matter, %	90.00		Cysteine	0.191
Crude Protein, %	18.03		Methionine + Cysteine	0.497
Ethereal Extract, %	4.02		Lysine	0.894
Fiber in Neutral Detergent, %	47.50		Threonine	0.797
Fiber in Acid Detergent, %	26.23		Arginine	0.928
Mineral Matter	10.66		Isoleucine	0.769
Total Phosphorus, %	0.402		Leucine	1.433
Available Phosphorus^2^, %	0.132		Valina	0.922
Calcium, %	1.810		Histidine	0.363
			Phenylalanine	1.078
			Glycine	0.940
			Serina	0.793
			Proline	0.794
			Alanine	1.027
			Aspartic acid	1.593
			Glutamic acid	1.926
			Digestible amino acids^4^, %	
			Lysine	0.599
			Methionine + Cysteine	0.338
			Methionine	0.241
			Threonine	0.502
			Arginine	0.622

^1^ Kakengi et al. [11]

^2^ value determined considering the availability of total phosphorus of plant origin of 33%

^3^ estimated values according to amino acid analysis carried out by the company Evonik Industries AG

^4^Estimated values considering digestibility of 68, 79, 68, 63, and 67%, respectively, for the alfalfa amino acids Lysine, Methionine, Methionine + Cystine, Threonine, and Arginine [32].

Leaf flour samples were also analyzed for the presence of antinutritional compounds at the Protein Biochemistry Laboratory belonging to the Federal University of Pernambuco, where hemagglutinating activity (lectin) and trypsin inhibition were verified. To carry out the analyses, initially the leaf flour was subjected to protein extraction at 10% (w/v), under constant agitation for 16 hours, in 0.15M NaCl saline solution, subsequently, the entire content was filtered through paper filter and subjected to centrifugation at 8,000 rpm for 20 minutes to obtain the crude extract.

After obtaining the extract, the hemagglutinating activity (HA) was determined according to the methodology proposed by Correia and Coelho [31] by adding 50μl of 0.15M NaCl to all the wells of a microtiter plate consisting of 8 rows of 12 wells each. Skipping the first well (control) of the horizontal row, 50 μl of the crude leaf flour extract, followed by successive dilutions, were placed in the following wells, discarding the final 50 μl.

Subsequently, 50 μl of the rabbit erythrocyte suspension was added to each well and the plate remained at rest for a period of 45 minutes, at room temperature, with the hemagglutinating activity defined as the inverse of the highest titer, in which an agglutination different from the control is observed.

The extract was also analyzed for protein concentration, according to the methodology of Lowry et al. [33]. The soluble protein content in the extract was used to calculate the specific activity of trypsin inhibitors.

Trypsin inhibition activity was evaluated according to the methodology described by Pontual et al. [34], in which 96-well microtiter plates were used, using 0.1 mg/ml bovine trypsin in 0.1 M Tris-HCl at pH 8.0 containing 0.02 M CaCl2. 5 μl of bovine trypsin was incubated for 5 minutes at 37°C, and for 30 minutes with crude moringa flour extract (50 μl) in Tris-HCL buffer at pH 8.0.

Later, the synthetic substrate BAPNA was dissolved in dimethyl sulfoxide (5μl) and the mixture was incubated for 30 minutes at 37°C. Then, substrate hydrolysis was followed by absorbance measurement at 405 nm.

Trypsin inhibition activity (UIT) is defined as the number of trypsin units inhibited per mg of sample. The specific inhibition activity was obtained considering the protein content of the extracts and was expressed as UIT/mg of protein.

### Experimental design and diets

The birds were distributed in a completely randomized design (DIC), with five treatments, including diets with 0; 1.5; 3; 4.5 and 6% moringa leaf meal and five replicates of six birds each.

The composition of the feed and the nutritional requirements of the birds used to formulate the diets followed the recommendations proposed by Rostagno et al. [35], with the exception of the metabolizable energy contents of corn, soybean meal, and soybean oil, which used the values proposed by Silva et al. [36], at 3853, 2753, and 8314 kcal/kg, respectively. For the formulation of the diets, the composition of *Moringa oleifera* analyzed at the Animal Nutrition Laboratory (LNA) at UFRPE, previously mentioned in Table 1, was considered.

The percentage composition and nutritional levels of the experimental diets are represented in Table 2.

**Table 2:** Percentual and bromatological composition, and nutritional levels of experimental feed.

	**Níveis de Moringa %**				
Ingredients, %	0	1.5	3.0	4.5	6.0
Ground corn	61.25	60.80	60.35	59.90	59.45
Soybean meal	23.53	22.94	22.34	21.74	21.15
Calcitic limestone	10.08	10.02	9.95	9.89	9.83
*Moringa oleífera*	0.00	1.50	3.00	4.50	6.00
Soybean oil	1.66	1.66	1.66	1.66	1.66
Dicalcium phosphate	1.03	1.04	1.05	1.06	1.06
Common salt	0.32	0.32	0.32	0.32	0.33
Sodium bicarbonate	0.25	0.25	0.25	0.25	0.25
DL-methionine 99%	0.17	0.17	0.18	0.18	0.18
Inert	1.60	1.20	0.80	0.40	0.00
Vitamin and mineral premix^1^	0.10	0.10	0.10	0.10	0.10
**Total**	**100.00**	**100.00**	**100.00**	**100.00**	**100.00**
**Calculated and analyzed levels**					
Metabolizable energy (kcal/kg)	2,800	2,800	2,800	2,800	2,800
Crude protein (%)	16.00	16.00	16.00	16.00	16.00
Crude protein analyzed (%)	16.85	16.26	16.29	16.00	16.06
Dry matter ^2^, (%)	90.75	91.39	91.48	91.48	91.28
Total Crude Fiber (%)	2.44	3.06	3.68	4.23	4.91
Neutral detergent fiber^2^ (%)	23.66	25.77	25.28	25.65	25.45
Acid detergent fiber^2^ (%)	6.06	6.51	7.19	7.59	8.08
Mineral matter^2^ (%)	16.59	16.43	17.47	16.00	15.81
Ethereal extract^2^ (%)	3.90	4.32	4.88	3.86	4.09
Digestible Methionine + Cystine (%)	0.634	0.634	0.634	0.634	0.634
Digestible methionine (%)	0.391	0.393	0.396	0.399	0.402
Digestible lysine (%)	0.729	0.724	0.718	0.712	0.707
Digestible tryptophan (%)	0.167	0.167	0.167	0.168	0.168
Arginine (%)	0.967	0.958	0.949	0.939	0.930
Calcium (%)	4.200	4.200	4.200	4.200	4.200
Available phosphorus (%)	0.291	0.291	0.291	0.291	0.291
Sodium (%)	0.218	0.218	0.218	0.218	0.218
Potassium (%)	0.613	0.613	0.613	0.613	0.613
Chlorine (%)	0.235	0.235	0.234	0.234	0.234
Fat (%)	3.96	3.95	3.92	3.90	3.88
Linoleic acid (%)	2.25	2.24	2.22	2.21	2.19

^1^Guarantee levels per kg of product: Folic acid (min.) 200mg; pantothenic acid (min.)5,350 mg; copper (min.) 4,000 mg; iron (min) 20 g; iodine (min.) 1,500 mg; manganese (min.) 75 g; niacin (min.) 19.9 g; selenium (min.) 250 mg; Vit. A (min.) 8,000,000 IU; Vit. B12 (min.) 10,000 mcg; Vit. B2 (min.) 4,000 mg; Vit. B6 (min.) 1,000 mg; Vit. D3 (min) 2,000,000 IU; Vit. E (min.) 15,000 IU; Vit. K3 (min.) 2,000 mg; zinc (min.) 50 g. ^2^ Values analyzed at the UFRPE Animal Nutrition Laboratory.

### Parameters evaluated

#### Performance

The parameters evaluated were feed intake (g/bird/day), average egg weight (g), egg mass (g/bird/day), laying percentage (%), feed conversion per dozen eggs (g/dz) and per egg mass (g/g).

Feed intake (FI) was determined by dividing the difference between the feed provided during the treatment phase and the leftover feed weighed at the end of the phase by the number of birds in the plot, and then calculated to obtain the average FI per bird in the plot (g/bird/day).

Eggs were collected twice a day (8:00 a.m. and 3:00 p.m.) and recorded on a laying frequency and mortality form for data correction. Percentage of egg production was obtained by collecting the number of eggs produced daily corrected for mortality, so that the ratio of whole eggs produced was expressed as a percentage for each treatment, over the average number of birds in the period (%/bird/day). This corresponded to the production of marketable eggs.

All whole eggs produced in the last three days of each production cycle were weighed individually by using analytical scales (0.001 g) to obtain the average egg weight, which was then multiplied by the total number of eggs produced in the experimental period, thus obtaining the total egg mass. This mass was divided by the total number of birds per day, and expressed in grams of egg/bird/day.

Feed conversion per egg mass (g/g) was calculated by the ratio of feed consumption to egg mass produced. Conversion per dozen eggs (g/dz) was calculated by dividing feed consumption by egg production, with the result then multiplied by twelve.

#### Evaluation of egg quality parameters

On the last three days of each cycle, three eggs produced per experimental plot were evaluated for egg quality parameters: egg weight (g), specific gravity (g/ml), yolk color (score), height of albumen (mm), yolk weight (g), shell thickness (mm), shell weight (g), albumen weight (g) and percentage of yolk, albumen and shell (%). Before starting the analysis, all eggs were identified according to the treatment and their respective repetitions.

After that, the eggs were sent for analysis to the Meat Laboratory of the Department of Animal Science at UFRPE, where they were weighed individually on a precision scale with a variation of 0.01g (Bel, model L 3102iH). After weighing, all eggs were immersed in a saline solution with densities ranging from 1.050 to 1.100 g/cm^3^, with an interval of 0.05, to evaluate specific gravity, according to the methodology proposed by Card and Nesheim [37], which used a petroleum hydrometer with a scale of 0.05 for calibration.

Then, eggs were broken individually on a flat surface, and the height of the albumen was measured with the aid of a digital caliper at a scale of 0.01 mm. Once the measurement was carried out, the albumen was manually separated from the yolk and the yolk was weighed individually on a precision scale. Immediately after weighing the yolks, color analysis was carried out according to the methodology described by Galobart et al. [38] by using a DSM® colorimetric fan with a color scale of 1 to 15. Color analysis was carried out by three different evaluators who assigned color scores to the gems.

The shells were carefully washed and left to air dry for a period of 48 hours. After this drying period, the shells were weighed on a precision scale and their thickness was measured with a digital caliper.

With the data obtained, the Haugh Unit [39] of the treatments was calculated by using the formula proposed by Card and Nesheim [37]. Where: UH = 100 log (H+7.57–1.7W0.37), H is the height of the albumen (mm), and W is the weight of the egg (g).

### Serum parameters

At the end of the experimental period, two birds per plot (10 birds per treatment) were selected at random and 4 ml of blood was drawn from the left-wing vein of each bird. The blood was stored in a dry tube with a clot activator (BD Vacuteiner® Seco), and after 30 minutes it was centrifuged at 3,500 rpm in a centrifuge (SL-702/RAF30, Solab) for 1 minute to obtain the serum, which was later stored in 2 ml Ependorf tubes and frozen for posterior analysis.

Subsequently, all serum samples were subjected to analyses of total cholesterol (Bioclin Kit, Reference k083-2), triglycerides (Bioclin Kit, Reference k117-2), albumin (Bioclin Kit, Reference k040-1), uric acid (Bioclin Kit, Reference k139-2), and total proteins (Bioclin Kit, Reference k031-1), by using commercial kits (Bioclin) in accordance with the manufacturer’s technical guidelines. Spectrophotometry equipment was used to read the parameters, and these analyses were carried out at the Laboratory of Molecular Biology Applied to Production (BIOPA/UFRPE).

### Statistical analyses

The blood parameter data were subjected to the Bartlett test to evaluate the homogeneity of the data and to logarithmic transformation. Blood parameter data and other data obtained were subjected to analysis of variance. The means were compared by the Dunnet test, and when significant (P<0.05), subjected to regression analysis by using the Statistical Analysis System version 9.4, SAS Institute Inc., to verify the effect of inclusion levels on the results.

## Results and discussions

The results of hemagglutinating activity and trypsin inhibitor activity analyses revealed values of 0.446 AHE (specific hemagglutinating activity) and 0.420 UTI/g (inhibited trypsin unit).

According to the results of these analyses, it can be observed that moringa leaf flour shows small concentrations of anti-nutritional factors, which agrees with data reported by Teixeira [19] when determining the chemical and nutritional characteristics of moringa leaves and verified trypsin inhibition values of 1.45 UTI/g of sample. The trypsin inhibition values reported by Teixeira [19] may possibly be related to the age of the plant used, considering that the leaf samples came from older plants used for afforestation. The plants used in this research had a physiological age of 45 days, which possibly provided plants with lower concentrations of these compounds.

As with trypsin inhibitors, positive results were also observed for hemagglutinating activity (AHE), indicating the presence of lectin in the leaf flour. However, the concentrations observed were greatly reduced when compared to the activity observed in moringa seeds by Coelho et al. [40], with values ranging from 100 to 2598 AHE. Makkar and Becker [41] did not detect the presence of trypsin and lectin inhibitors in moringa leaves. Later Ferreira et al. [42] reported that trypsin and lectin inhibitors are absent in *Moringa oleifera* leaves.

Despite being contradictory to those previously reported, the data obtained show the existence of antinutritional factors in moringa leaves. Despite being present, their concentrations are quite low because possibly they are not enough to cause significant effects on performance, which allows for their use in animal feed.

The results of the performance parameters obtained during the experimental period (Table 3) demonstrate that the inclusion of different levels of moringa leaf meal in the diet was well accepted by the birds, and did not influence the parameters feed intake, feed conversion per mass, and per dozen eggs produced, number and mass of eggs, and laying percentage. When considering the results obtained for the regression analysis, it can be observed that moringa did not have a significant effect on the productive performance parameters, demonstrating that its incorporation in the feed of laying hens is possible up to the maximum level analyzed (6%) without causing harm to birds.

**Table 3:** Performance of laying hens fed different levels of *Moringa oleifera* in the diet during the period from 62 to 77 weeks of age.

	MORINGA LEVELS								
VARIABLES	0.0	1.5%	3.0%	4.5%	6.0%	P	DP	CV	R^2^
Feed intake (g/bird/day)	97.85	97.44	99.30	97.38	97.47	0.83	5.97	6.10	-
Feed conversion per egg mass (g/g)	1.930	1.870	1.929	1.887	1.894	0.60	0.14	7.52	-
Feed conversion per dozen eggs (g/dz)	1.454	1.446	1.459	1.441	1.465	0.99	0.16	11.00	-
Average egg weight (g)	58.17^b^	59.77^b^	59.63^b^	60.73^a^	60.83^a^	0.00	2.16	3.61	-
Egg mass (g/bird/day)	50.89	53.20	51.94	53.01	53.80	0.41	3.74	7.15	-
Laying percentage (%)	87.61	87.65	88.64	88.21	86.86	0.90	5.96	6.79	-

P: Probability, DP: Standard deviation, CV: Coefficient of variation, R: regression, NS: Not significant. Numbers followed by the same letters do not differ significantly by the Dunnet test at the 5% probability level

A similar response was also observed by Kakengi et al. [11] who found no alterations in feed intake, feed conversion, egg mass, and laying percentage when replacing sunflower seed meal by 5% with moringa leaf flour in the diet of Leghorn laying hens from the 20^th^ to the 33^rd^ week of age.

According to Olugbemi et al. [43], Paguia et al. [44], and Gakuya et al. [45], moringa is well tolerated in the diet of laying birds at levels of up to 10% without causing a significant reduction in feed intake and bird performance. This greater acceptability of diets with moringa leaves by birds compared to other forages, such as Gliricidia Sepium, may be related to the lower concentration of antinutritional compounds (condensed tannins, saponins, trypsin inhibitor, and lecithin) [14, 41, 42], which could act on the digestibility of its nutrients, as well as act as an astringent factor directly influencing intake.

Odunsi et al. [46] found a significant reduction in feed intake from the lowest level of inclusion with a significant reduction in bird weight when evaluating the addition of Gliricidia Sepium leaf meal to the feed of laying hens (Harco) at different levels (5, 10, 15%), which was justified by high levels of anti-nutritional factors and low palatability of the forage.

The experimental diets used in this research were formulated considering the nutritional contribution of all ingredients, including moringa, in order to meet the needs of the birds and yield the same levels of nutrients and energy (isonutritive and isocaloric). Thus, the results obtained in the productive performance of the birds demonstrate that the moringa leaf meal used in the diets met the birds’ needs, since it did not negatively influence performance.

On the other hand, the average weight of eggs was significantly different when submitted to the Dunnett test, which confirmed the influence of moringa levels on this parameter when compared with the control treatment. However, when subjected to regression analysis, no significant responses were obtained. An increase in egg weight was observed for levels of inclusion of 1.5%, 4.5%, and 6%, when compared to the control treatment. Although not significant, it was observed that the 3% inclusion level favored an increase in egg weight, although not enough to be identified in the tests applied.

Similar results were observed by Ebenebe et al. [12] when evaluating different levels of moringa inclusion (0, 2.5%, 5% and 7.5%). They obtained an increase in the production and weight of eggs of Isa Brown layers (16 to 24 weeks of age) in relation to the control group when providing 2.5% moringa in the diet. They also observed significant improvements in the average weight of eggs in breeding birds from 22 to 34 weeks of age with the inclusion of 5% *Moringa oleifera* in the diet [47].

However, research such as that by Gakuya et al. [21] using inclusion levels of 1.25%, 2.5%, 5%, 7.5%, and 10% of leaf meal, reported no influence of moringa on production parameters and average egg weight when they used Isa Brown layers from 30 to 34 weeks of age. However, it can be observed that the experimental period observed was only four weeks, and possibly this period was insufficient to obtain any effect from the dietary treatments.

In general, significant increases in the average weight of eggs are observed when using levels of moringa inclusion between 5 and 10%. Above these levels, the average weight of eggs tends to decrease, as well as promote worsening in productive parameters [8, 11, 48].

The responses observed for the average weight of eggs with the use of *Moringa oleifera* in layer rations are not yet well understood. As a result, some authors have reported theories that could be related to the increase observed for this parameter. According to research developed by Hassan et al. [49], *Moringa oleifera* improves protein digestibility and nutrient utilization because it contains flavonoids that react as antimicrobial and antioxidant agents. This same author reported that moringa may have a beneficial effect on the microbial environment of the intestine, which would improve digestion, absorption and use of nutrients.

Research has shown that *Moringa oleifera* has antibacterial properties [50, 51], due to the presence of phytochemical compounds such as saponins, tannins, phenols, and alkaloids, which act on bacteria of the *Salmonella tiphimurium* and *Salmonella enteritis* genus [52], which respectively cause food poisoning in humans, and colonize the egg-laying canal of birds causing contamination of the membrane surrounding the yolk during egg formation, and leading to loss of productivity, increased mortality, and contamination of poultry products [52].

This antimicrobial action has also been attributed to the substance 4(α-L-rhamnosyloxy) benzyl isothiocyanate, found in the leaves and mainly in the seeds of *Moringa oleifera* [53, 54].

Antimicrobial compounds, when present in poultry feed, reduce the proliferation of bacteria that cause negative effects on intestinal health, and provide an improvement in the digestive process, since the integrity of the intestinal mucosa where enzymatic digestion and absorption of nutrients happens, is maintained [55].

Another theory that could explain the observed increase in egg weight may be related to the presence of fiber in the birds’ diet. The inclusion of *Moringa oleifera* leaf flour increased dietary NDF levels by 0.45; 1.13; 1.53 and 2.02% respectively for levels between 1.5% and 6% of inclusion. According to Hetland et al. [56], birds have a requirement for fiber to stimulate the anterior digestive tract, considering that the structural components of the diet increase digesta reflux, possibly caused by increased gizzard activity. These movements facilitate contact between nutrients and endogenous enzymes [57].

The combination of greater gizzard work associated with intestinal movements and greater contact time between dietary components and digestive enzymes may have favored greater protein and energy digestibility, which were directed towards egg development.

In more recent research, Teteh et al. [58], when evaluating the inclusion of levels of 1 and 2% of leaf flour in diets for Isa Brown layers from the initial phase (1 day of life) to production (40 weeks of age), verified higher egg weights with inclusion of 1% of moringa and an increase in the relative weight of reproductive organs (ovary and oviduct) and a greater number of follicles. They attribute this response to the presence of phytosteroids present in moringa leaves. Moringa leaves contain phytosteroids (stigmasterol, sitosterol, and kampesterol) in their composition, which are used as a precursor for the synthesis of sexual steroids, including estrogen [59].

Therefore, estrogen could have influenced the relative weight of the oviduct, as well as having an effect on the tubular glands and epithelial cells of the mucosa and magnum, where albumin production occurs [58]. Thus, according to this same author, with oviduct development sexual hormones could have induced the group fed with 1% leaf flour to increase albumen production, which is responsible for increasing egg weight. The 2% level of inclusion may have provided a high level of estrogen, resulting in impaired development of reproductive organs.

When considering the theory reported by Teteh et al. [58], it can be assumed that the birds in research already had a developed reproductive system and the presence of these phytosteroids may have influenced albumen production, which despite not showing a significant response among the treatments, an increase in albumen weight was observed (Table 4) depending on the levels of Moringa leaf meal inclusion in the birds’ diet.

**Table 4:** Egg quality parameters of Dekalb White laying hens fed with different levels of *Moringa oleifera* leaf meal in the diet.

VARIABLES	Levels of Moringa %								
	0	1.5	3.0	4.5	6.0	P	DP	CV	R2
Egg weight (g)	58.17^b^	59.77^b^	59.63^b^	60.73^a^	60.83^a^	> 0.00	2.160	3.61	-
Yolk weight (g)	14.53	14.54	14.97	14.62	15.30	0.13	1.117	7.55	-
Albumen weight(g)	38.28	39.24	39.08	40.12	39.99	1.15	1.571	3.94	-
Albumen height (mm)	6.68^b^	6.78^b^	6.49^b^	6.38^a^	6.40^b^	> 0.00	0.372	5.69	-
Shell weight (g)	5.41^b^	5.54^b^	5.55^b^	5.69^a^	5.63^a^	> 0.00	0.244	4.39	-
Shell thickness (mm)	0.38	0.38	0.37	0.37	0.37	0.05	0.016	2.34	-
Specific gravity(g/cm^3^)	1.084	1.083	1.083	1.086	1.083	0.49	0.005	0.49	-
Yolk color (1–15)	5.00^b^	5.75^a^	6.62^a^	7.05^a^	7.54^a^	> 0.00	0.736	11.52	0.97 L
Haugh Unit	81.64^b^	81.84^b^	80.09^b^	78.69^a^	79.68^a^	> 0.00	2.259	2.81	0.96 Q
	Proportion of egg components %								
Yolk	25.08	24.45	25.11	24.86	25.07	0.079	0.836	3.36	
Albumen	65.58	66.27	65.64	65.81	65.64	0.1557	0.967	1.47	
Shell	9.36	9.29	9.30	9.30	9.28	0.972	0.359	3.86	

Numbers followed by the same letters do not differ significantly according to the Dunnet test at the 5% probability level. 0.97: Linear regression equation (L).; 0.96: Quadratic regression equation (Q). L: Y = 0.3867X + 5.29; Q: Y = 0.3044X^2^ - 2.08087X + 85.47.

To date, some research has been reported on the use of moringa in feeding laying birds, with significant responses in egg weight [8, 11, 12, 47, 48, 58, 60]. However, as already mentioned, it is not yet clear what causes this increase, whether it is caused only by an isolated factor or by the association of both factors previously reported.

Table 4 shows the data obtained for egg quality. Among the parameters analyzed, there was no influence of moringa levels for specific gravity, yolk weight, albumen weight, shell thickness and for the proportions of yolk, albumen, and shell.

When considering the results of the Dunnet test, the influence of treatments on shell weight, albumen height, Haugh Unit, and yolk color is observed. However, when the data were subjected to regression analysis, it was observed that the inclusion of *Moringa oleifera* leaf flour significantly influenced the parameters of yolk color and albumen height. For the other parameters analyzed, no significant effects were observed due to the inclusion levels.

Significant differences were observed for eggshell weight among control treatments and inclusion levels of 4.5 and 6%. The data obtained were similar to those found by Ebenebe et al. [12], which showed an increase in eggshell weight with the inclusion of 2.5% and 5% of moringa in the diet of 24-week-old Isa Brown birds.

Such results were also reported by Tesfaye et al. [47], who found that the inclusion of 5% *Moringa oleifera* associated or not with cassava peel provided heavier eggshells and obtained higher values than the control group (0% cassava peel and moringa).

In more recent research, Kana et al. [61] did not obtain significant differences for eggshell weight when evaluating the effect of replacing soybean meal with *Moringa oleifera* leaf flour on the quality characteristics of eggs from Kabir hens. They obtained values ​​that corresponded to 4.69, 4.55, and 4.67g, for levels of 0 (control), 50, and 100% replacement.

Although the increase in eggshell weight has not previously been justified by other authors, it is assumed that such an increase may be related to the increase in egg weight.

Birds have a limited capacity for calcium deposition in the eggshell. Due to the increase in egg size, a similar amount of calcium has to be distributed over a larger surface [62]. Consequently, larger eggs have greater shell weight when compared to smaller eggs. However, despite the greater shell weight, it was observed that the shell thickness tended to decrease (P = 0.05), which ends up directly influencing the resistance of the eggs.

Although significant differences were observed for egg and shell weight, these differences did not provide variation in the proportion of egg components, which remained similar in all treatments.

**Fig 1:**
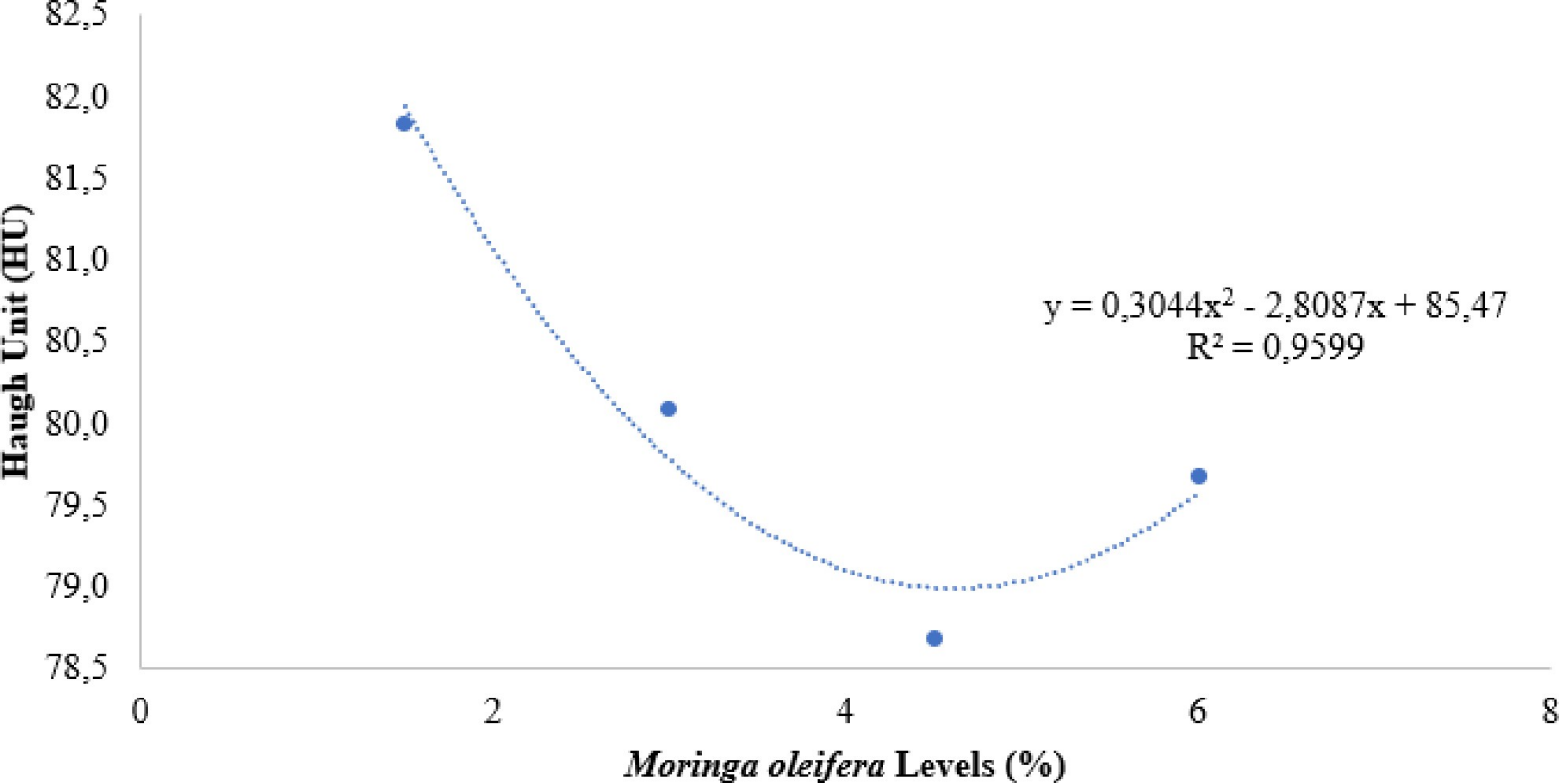
Haugh unit of eggs from commercial layers fed with different levels of *Moringa oleifera* in the diet.

The results obtained for the Haugh Unit showed quadratic behavior (Fig 1) according to the levels of moringa in the diet, negatively influencing the values of this parameter. With the derivation of the equation, it was found that the level of moringa inclusion that generated the lowest HU values was 4.61%.

The birds submitted to diets containing *Moringa oleifera* levels of 4.5 and 6% significantly reduced Haugh unit values when compared to the control group. The Haugh unit is a measurement that correlates egg weight with albumen height. Thus, it was clear that the Haugh unit values obtained were proportional to the reduction in albumen height, which despite being significant only for the 4.5% inclusion level, it appears that among the other levels there is a decline.

The albumen has three layers, one closest to the yolk (fluid), which is then surrounded by another intermediate layer (thicker albumen), also known as the albuminous sac, and finally another outer layer of fluid albumen [63].

As reported, the eggs showed an increase in weight, according to the levels of moringa in the diet and, consequently, a reduction in HU values. This increase may have occurred due to an increase in albumen, more precisely fluid albumen, which contributed positively to the weight of the egg but not to the height of the albumen, since only the dense albumen portion is considered for this measurement.

Research that justifies the reduction of the Haugh unit with the use of *Moringa oleifera* in the diet of layers is still scarce, although Tesfaye et al. [47] developed research including 5% *Moringa oleifera* for 22-week-old laying hens and did not identify any influence of treatments on Haugh unit values.

When considering the use of other forages used to feed laying birds, different responses for this parameter are identified. Laudadio et al. [64] found no significant differences for the Haugh unit when investigating the effect of alfalfa hay bran on the productive parameters of Isa Brown laying hens (18 weeks old) and on egg quality.

Although Al-kirshi et al. [7] investigated the inclusion of mulberry hay (Morus alba) in the diet of Isa Brown laying hens (26 weeks old) at different levels (0, 10, 15, and 20%) and found a significant increase in the Haugh unit of eggs, higher values were observed for the highest level of inclusion of mulberry flour when compared to the control diet (0%), which demonstrated values of 91.1 and 76.7 units, respectively.

According to Lemos et al. [65] the United States Department of Agriculture (USDA) classifies eggs into four groups based on the Haugh unit (HU), as follows: excellent: > 72 HU; good quality: 60–72 HU; medium quality: 55–30 HU and low quality: < 30.

The Haugh unit results obtained for the highest levels of inclusion are within the classification standards cited by Lemos et al. [65]. Although the unit values have been reduced, they are still within the classification of eggs as being of excellent quality, and had values of 78.69 and 79.68 HU, for the respective levels of 4.5 and 6% of inclusion of moringa leaf meal.

**Fig 2:**
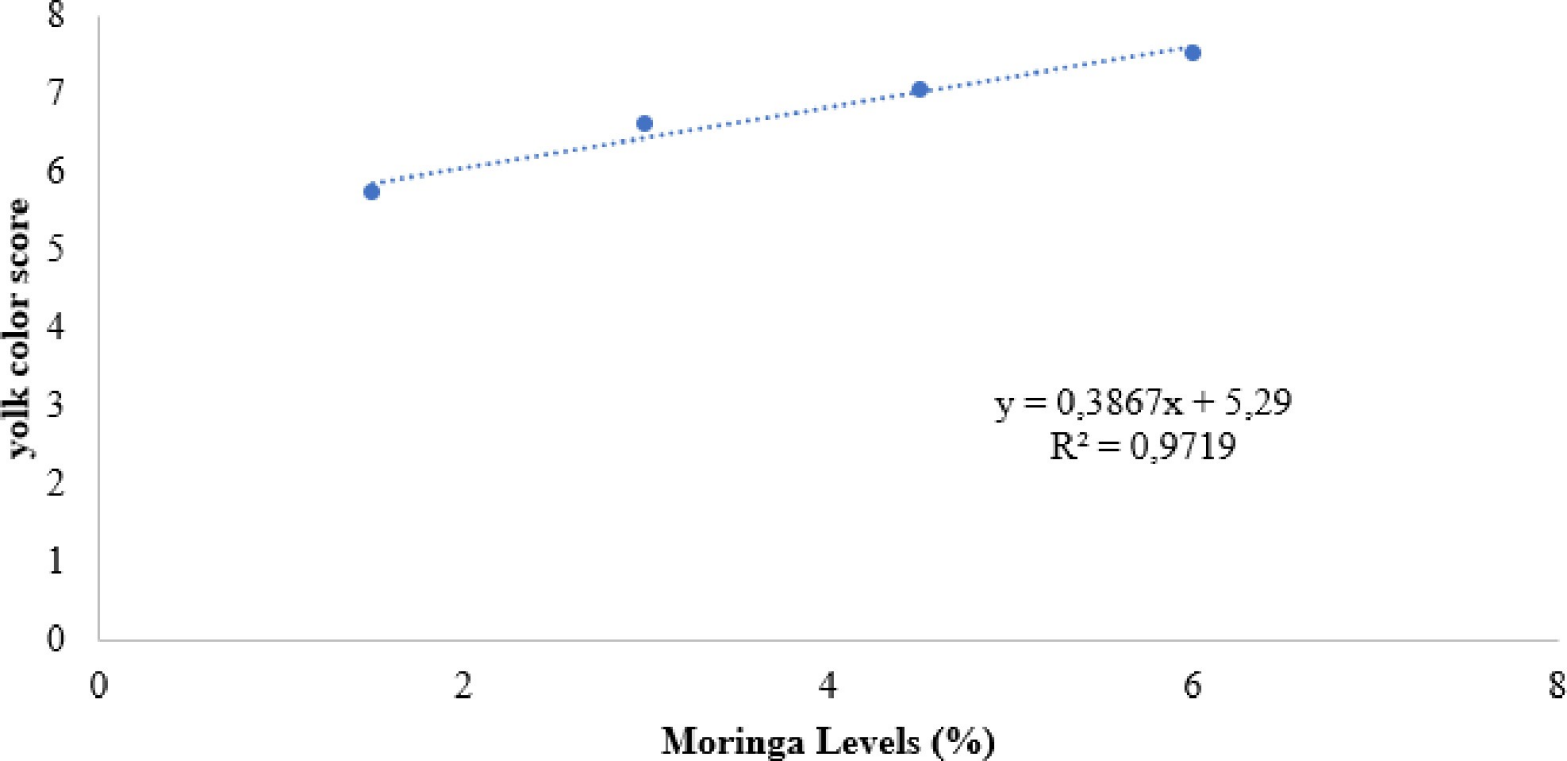
Effect of *Moringa oleifera* levels on the egg yolk color of eggs from commercial laying hens.

The results obtained for yolk color demonstrated linear growth in color according to the levels of moringa inclusion in the birds’ diet (Fig 2). When the data were subjected to the Dunnett test, it was possible to verify that the inclusion of moringa provided increases in the pigmentation of the yolks among the different levels of inclusion (1.5 to 6%), providing significant increases of 15, 32.4, 41, and 50.8%, respectively for the levels of 1.5%, 3%, 4.5%, and 6%.

Birds are not capable of synthesizing pigments, but they have the ability to transport those ingested in the diet to the yolk, thus, the color of the yolks reflects the carotenoid profile of the diet [66].

These observations were also reported by Olugbemi et al. [43] when evaluating inclusion levels of *Moringa oleifera* of 5 and 10%, and cassava peels for laying hens. With the addition of moringa to the birds’ diet, color scores increased from 1.24 and 1.15 (diets without added moringa) to 6.05 and 7.79, respectively, for inclusion levels of 5 and 10%.

Linear response in egg yolk color was also observed by Abou-Elezz et al. [67] when using inclusion levels of 5, 10, and 15% of moringa leaf flour for Rhode Island Red chickens. They obtained color scores of 8.25, 9.92, and 11.00 for the respective inclusion levels. When *Moringa oleifera* is used in poultry feed at levels greater than 5%, there is no need to include artificial colors [21].

In addition to hay, supplementation with fresh forage also provides significant improvements in the color of egg yolks, without affecting the productive performance of the birds [67].

The intensification of egg yolk color is associated with the high concentrations of carotenoids present in moringa leaves, equivalent to 16.3 mg/100g of leaves “NRC, 1985” apud Abou-Elezz et al. [67]. Research developed by Moyo et al. [17] and Tesfaye et al. [47] demonstrated β-carotene concentrations of 18.5 and 15.25 mg/g DM, respectively.

More specifically, the improvement in color comes from the concentrations of xanthophylls (yellow and red pigments) present in moringa leaves [48]. Carotenes are hydrocarbons, and due to this characteristic, they have a reduced capacity for deposition in the egg yolk. Xanthophylls (oxycarotenoids) have oxygenated radicals (hydroxyl, ketone, or ester) that allow for more efficient deposition. Among the main oxycarotenoids, lutein, zeaxanthin, canthaxanthin, citranaxanthin and capsanthin stand out [68]. Moringa is rich in biologically active xanthophylls and can improve the color of poultry products and reduce the costs of synthetic pigments [69].

According to Laudadio et al. [64], higher concentrations of these pigments in bird feed result in a greater concentration in the egg yolk, directly influencing color. Although yolk color is not associated with better nutritional value, freshness and cooking characteristics are main parameters by which egg quality is judged by the consumer [70].

Considering blood biochemistry parameters, there was no significant difference among the treatments for the levels of total cholesterol, urate, albumin and total proteins (Table 5).

**Table 5:** Blood parameters of commercial layers fed with different levels of *Moringa oleifera* leaf meal from 62 to 78 weeks of age.

	Inclusion levels							
Parameters	0	1.5	3.0	4.5	6.0	CV	P	DP
COL (mg/dl)	99.09	142.9	117.44	138.92	86.07	8.11	0.419	0.165
PT (g/dl)	20.04	20.75	20.16	17.66	15.28	10.67	0.468	0.133
ALB (g/dl)	3.33	3.10	3.39	3.63	2.85	14.77	0.366	0.074
UR (mg/dl)	4.75	5.55	5.26	4.95	5.27	11.07	0.820	0.078

COL- Cholesterol; PT- Total proteins; ALB- Albumin; UR- Urate.

According to Stringhini 1998, apud Jardim Filho et al. [71], measurements of blood parameters, such as uric acid, albumin, total proteins, and glucose, are good indicators of the bird’s nutritional condition in terms of proteins and minerals. Although a large number of repetitions are necessary, since the coefficient of variation of these analyses is usually high.

The biochemical parameters of the blood of birds fed with moringa were not altered depending on the levels in the diet, demonstrating that the needs of the birds were met at both levels evaluated.

Although the results obtained do not demonstrate significant responses, some research using moringa leaf flour has demonstrated an influence on the serum responses of some parameters.

Among the parameters described is cholesterol, which at the inclusion level of 6% presented lower values than the control group, with respective values ​​of 86.07 and 99.09 mg/dl. According to Olugbemi et al. [48], plasma cholesterol levels in layers were reduced when 5 and 10% levels of moringa leaf flour were included in the birds’ diet over a period of 90 days. Reductions in serum cholesterol levels were also reported by Elkloub et al. [72], when they included moringa leaf flour in the diet of Japanese quails during an experimental period of 42 days.

On the other hand, Tesfaye et al. [47] found no significant response to total cholesterol levels in laying hens when replacing soybean meal by 5% with moringa leaf flour in the birds’ diet between the 22nd and 34th week of age.

Non-significant responses were observed in this research carried out despite some studies mentioned above demonstrating an effect on the serum levels of laying hens fed with moringa. Possibly, the different responses may be associated with the amount of moringa included in the birds’ diet. Despite the concentrations used in this research being above the minimum level assessed by Olugbemi et al. [48], they were not high enough to provide significant effects. However, it is important to highlight that numerically a decrease in the serum cholesterol levels of birds fed with an inclusion level of 6% was observed, which confirms the idea that the levels tested did not present significant amounts of photochemical compounds capable of causing effects on the blood parameters evaluated.

When considering the concentration of β-sitosterol, Rajanandh and Kavitha [73] revealed that moringa leaves have 90 mg of β-sitosterol per gram of sample. Evaluating the feed with the inclusion of 6% moringa and considering the concentration of β-sitosterol already reported, it can be estimated that on average the diet had 60 g of moringa per kilo of feed, and with this, it would be providing around 5400 mg of β-sitosterol per kilogram of diet. As the birds’ daily consumption was approximately 97 grams, it is estimated that the birds ingested around 523.8 mg of β-sitosterol per gram of feed. This concentration may have possibly contributed to the decrease in plasma cholesterol levels observed with the inclusion of 6% moringa in the birds’ diet.

## Conclusion

*Moringa oleifera* leaves can be used to feed commercial laying birds with up to 6% inclusion without causing harm to productive performance and egg quality, in addition to intensifying the color of the yolks.

## Supporting information

S1 File The values used to build graphs (Figures). (XLSX)
